# Health of school-age children and adolescents in Saudi Arabia: a systematic review

**DOI:** 10.1186/s12889-025-25897-x

**Published:** 2026-01-26

**Authors:** Ghadeer Aljuraiban, Reem F. Alsukait, Norah Alkanhal, Mariam M. Hamza, Amaal Alruwaily, Severin Rakic, Volkan Cetinkaya, Christopher H. Herbst, Ada Alqunaibet

**Affiliations:** 1 College of Applied Medical Sciences, King Saud University, Riyadh, Saudi Arabia; 2 Saudi Public Health Authority, Riyadh, KSA Saudi Arabia; 3 Health, Nutrition and Population Global Practice, The World Bank, Washington, D.C USA

**Keywords:** School health, Children, Adolescents, Risk factors, Morbidity, Mortality

## Abstract

**Background:**

Identifying the leading risk factors and major determinants of morbidity and mortality in school-age children and adolescents is pivotal for health and maintenance of well-being. This review provides an overview of health conditions/risk factors driving morbidity and mortality and identifies gaps in existing data to improve health and development needs in school-age children and adolescents.

**Methods:**

PubMed, Embase, Web of Science, and official websites of global databases and national surveys were searched from January 2012 until April 2022 for relevant publications reporting the prevalence of health conditions/risk factors that contribute to morbidity and mortality in children/adolescents (5–19 years of age) in the Kingdom of Saudi Arabia (KSA).

**Results:**

A total of 21 publications were included in the review. Overall, data on children aged 5–9 and 10–19 years old are very scarce except for health conditions assessed in the national school-based screening programs, and only one nationally representative study in 2011 for 10–19 year olds. Data for those ≥ 15 are more prevalent considering they are included in most nationally representative data (e.g., 2013 Saudi Health Interview, the 2018 Household Health, and the 2019 Sports) surveys. However, gaps remain. Overweight and obesity estimates for children and adolescents varied significantly across different datasets and data collection years, ranging from 37% to 18%, respectively, to 6% and 4% for overweight and obesity across different age groups. Mental health disorders were the leading cause of morbidity among children and adolescents aged 5–14-year-old accounting for 19% of the total burden. Road traffic injuries were the leading cause of death among children and adolescents aged 5–14-year-old at 25%.

**Conclusions:**

This review provides a snapshot of the health status of school-age children and adolescents in KSA and highlights the major data gaps especially for those < 10 year old where no nationally representative datasets exist. Many of the data sources for other age groups are not nationally representative or are too old to be useful for recommendations today. There is an urgent need to collect updated and robust data for school aged-children and adolescents in KSA to inform evidence-based policies and priorities. This study was not pre-registered in a publicly accessible registry.

**Supplementary Information:**

The online version contains supplementary material available at 10.1186/s12889-025-25897-x.

## Background

In 2018 there were about 8 million school-age children and adolescents (ages 5–19 years) in Saudi Arabia, making up about 23% of the population [1]. Childhood and adolescence are unique life stages in which behaviors and risk factors that may influence morbidity and mortality are formed or solidified [2]. These life stages represent a distinct opportunity to reinforce beneficial behaviors and remedy harmful ones.

Mortality rates in Saudi Arabia have been decreasing since 1990 among children and young adolescents aged 5–14 years, according to Global Burden of Disease (GBD) 2019 data [3]from 68 deaths per 100,000 in the 1990 s to 16 per 100,000 in 2019 [3]. This is lower than the global estimate of 52 per 100,000 total population. Despite the progress that has been made to reduce the mortality rate among children and adolescents in Saudi Arabia [4] and worldwide [5], they still account for about 6% of the burden of disease and injury [3, 5], with causes varying by region [2, 3, 5]. Recognizing leading risk factors and determinants for mortality and morbidity and intervening in these developmental age groups is essential for health and well-being and can set the stage to enhance health well into adulthood [2, 6, 7].

There are three phases in the health and development of school-age children and adolescents that require age-specific approaches [8]. First is the “middle childhood growth and consolidation phase” (ages 5–9), in which infection and malnutrition pose a crucial constraint to development, and mortality rates remain relatively high. Second is the “adolescent growth spurt” (ages 10–14), where body mass increases, and puberty is associated with significant physiological and behavioral changes. Last is the “adolescent growth and consolidation phase” (ages 15–19), in which the brain is restructured, there is experimentation and exploration, and behaviors that form life-long determinants of health are begun [8].

Schools are vital for realizing health promotion strategies in children and adolescents, especially since health has a clear link with educational outcomes [9, 10]. There are several reasons why schools are crucial locations: most people in these age groups are enrolled in schools, and much of their time is spent in schools, making this an ideal place for programs to reach them; schools are a space to teach skills and behaviors used later in life and can be a safe space for support and learning; and lastly, it can be used as a physical location to deliver health services [9, 11–14]. However, to best understand how to use the school environment to implement policies and emphasize health promotion, the current health status of students needs to be understood [9].

Government efforts to develop effective and evidence-informed strategies to meet the health and developmental needs of school-age children and adolescents in Saudi Arabia can be supported with a systematic review of evidence. This current systematic review presents an overview of the risk factors and health conditions contributing to morbidity and mortality in school-age children and adolescents in Saudi Arabia and highlights the gaps in data needed to improve the health and development needs of this age group.

## Methods

### Literature search

Two reviewers (GA and RS) conducted independent systematic searches using the electronic bibliography databases: PubMed, Embase, and Web of Science from January 2012 until April 2022. Included in the search were official websites of global databases and national surveys. Key words were searched both as individual search terms and Medical Subject Headings (MeSH) of the National Library of Medicine, e.g., exercise, physical activity, and fitness. Truncations and adjacencies were used to capture variations of the search terms and expand the reach of the search. To identify additional relevant publications, hand-searching of reference lists of published reviews was done. The detailed search strategy for PubMed is available in TableS1, where modified terms were used for other databases.

The identification of health conditions/risk factors contributing to morbidity and mortality in school-age children and adolescents was guided by indicators commonly measured in the Global School-based Student Health Survey of the World Health Organization (WHO) [15] and the Youth Risk Behavior Surveillance System of the Centers for Disease Control and Prevention (CDC) [16]. Priority was given to health conditions and risk factors typically addressed in school health–based national programs in Saudi Arabia (e.g., mental health) [17, 18] (Figure S1).

### Selection of studies

We included publications written in the English language and published between 2012 and 2022. Articles were observational studies reporting the prevalence of health conditions/risk factors that contribute to morbidity and mortality in children/adolescents (5–19 years of age) in Saudi Arabia. Publications with the most recent nationally representative data were prioritized in the selection. If national data were not available for a specific health condition/risk factor, then the most recent non-representative surveys were included. For outcomes with both national and non-national estimates, we reported the nationally representative estimate in the primary synthesis; when no national estimate existed, we reported the most recent eligible non-national estimate. Both self-reported and objective measures of the health condition/risk factors of interest were included. Excluded were: studies based on conditions outside the scope of this report (e.g., allergies), studies on the knowledge, attitudes, and practices of parents, healthcare providers’ opinions, and associations between the established risk factors, studies where the sample included preschoolers or college students and coping strategies during COVID-19, and publications in the grey literature, dissertations, commentaries, and conference proceedings. To exclude duplicates and screen records, the Covidence online software (Veritas Health Innovation Ltd, Australia) was used. Two reviewers (GA and RS) conducted an independent parallel screening of the title/abstract and full text. Disagreements that arose were resolved by consensus.

### Data extraction

Data extraction was completed by one reviewer and cross-checked for accuracy by a second reviewer. A standardized form that contained the following information was used: title of article, lead author’s name and publication year, aim of the study, location in Saudi Arabia, study design, year of data collection, characteristics of sample population, sample size, outcome measures, and main results.

### Risk of bias assessment

We assessed the risk of bias for included cross-sectional studies using the Newcastle-Ottawa Scale, a widely used tool for evaluating the quality of observational studies [19]. In brief, the scale evaluates studies across three domains: Selection (0–4 points), Comparability (0–2 points), and Outcome (0–3 points). Studies were then categorized as low risk of bias (7–9 points), moderate risk (5–6 points), or high risk (0–4 points), with a maximum total score of 9 points.

### Publication bias

We evaluated publication bias by visual inspection of funnel plots and by Egger’s regression test. For each outcome, we transformed study prevalences using the logit, calculated standard errors from binomial variance, and regressed the standardized effect on its precision. We considered a two-sided *p* < 0.05 as evidence of small-study effects. For every Egger test we report the intercept with its 95% confidence interval and p value. All funnel plots are provided in the Supplementary Figures, and the Egger statistics for every outcome are summarized in the Supplementary Tables.

## Results

### Results of the search

**Fig. 1:**
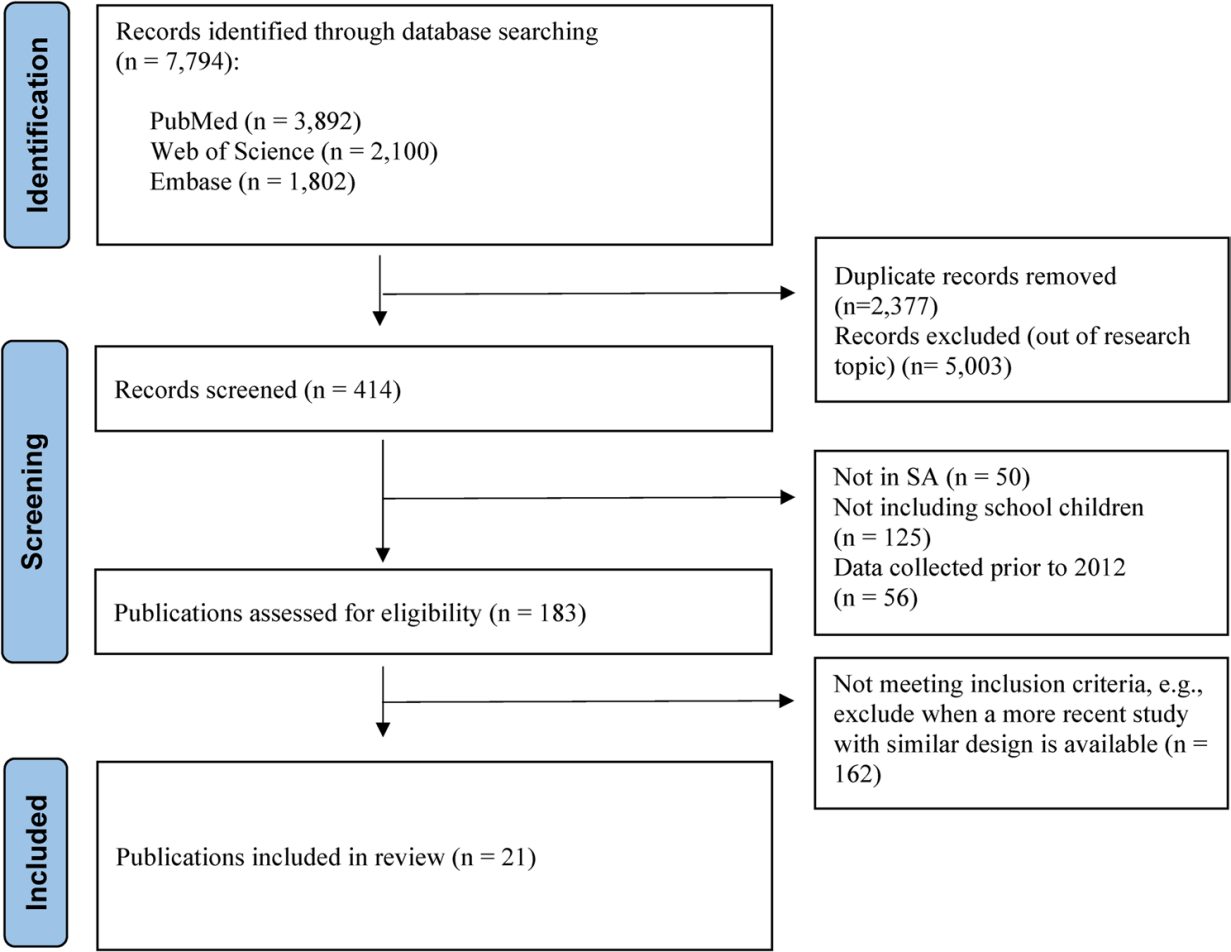
Flow diagram of identified records on health of school-aged children and adolescents in Saudi Arabia using the PRISMA diagram of search strategy and results for systematic reviews from: Page, M.J., et al., The PRISMA 2020 statement: an updated guideline for reporting systematic reviews. BMJ, 2021. 372: p. n71

Figure 1 shows the Preferred Reporting Items for Systematic reviews and Meta-Analyses (PRISMA) flowchart. Of the 7,794 records originally identified, the full text of 183 publications were reviewed. Out of these, 162 publications were subsequently excluded as they did not meet the selection criteria or were superseded by publications on the same topic that used national data. Finally, a total of 21 publications were included.

### Characteristics of included publications

Descriptive information on study design, year of data collection, location in Saudi Arabia, population, sample size, outcome measures, and main results are provided in Table 1. All studies included both boys and girls aged between 6 and 19 years, with sample sizes ranging from 317 to 552,579.

**Table 1:** Characteristics of publications of identified health conditions/risk factors

Reference	Study design	Year data collected	Location in Saudi Arabia	Study population (dataset)	Sample size	Outcome measures	Outcomes
AbouAbbas et al. [20]	Cross sectional	2011	13 regions of Saudi Arabia	Boys and girls, 10–12 y (Jeeluna study)	*N* = 12,121 (51% boys)	Self-administered questionnaire using the Youth Risk Behavior Surveillance System guided by the Diagnostic and Statistical Manual of Mental Disorders criteria for identifying underlying mental health disease	Sadness/depression, 14.3% (girls: 19% vs. boys: 10.1%); anxiety, 6.7% (girls: 9.1% vs. boys: 4.6%); older adolescents (> 15 y) reported feeling so sad or hopeless, 59%; worried, 66%; exposure to bullying at school during the preceding 30 d, 25.0% (girls: 22.7% vs. boys: 27.1%); involved in physical violence at school in the preceding year, 20.0% (girls: 11.7% vs. boys: 28.9%)
Al-Buhairan et al. [21]	Cross sectional	2011	13 regions of Saudi Arabia	Boys and girls, 10-18y (Jeeluna study)	*N* = 12,575 (51% boys)	Self-administered questionnaire using the Youth Risk Behavior Survey and the Global School-based Student Health Survey, anthropometric data measured, and blood samples collected	Diabetes, 0.7% (girls: 0.6% vs. boys: 0.9%); ever smoked cigarettes, 16% (girls: 9.6% vs. boys: 22.1%); sheesha (water pipe), 10.5% (girls: 7.1% vs. boys: 13.5%); solvent sniffing in the preceding month, 16% (girls: 21.4% vs. boys: 11.5%); fruit intake (≥ 1 servings/d), 38.0% (girls: 31.8% vs. boys: 43.6%); vegetable intake (≥ 1 servings/d), 54.3% (girls: 52.8% vs. boys: 50.7%); carbonated beverage consumption (≥ 2 drinks/d), 37.5% (girls: 30.4% vs. boys: 43.9%); energy drinks consumption (≥ 1 drinks/d), 21.8% (girls: 17.7% vs. boys: 25.5%); vitamin D deficiency (< 50 nmol/L), 95.6%; sometimes/always wear seatbelt, 13.8%; drive a car without permission 17.9%
Al-Daajani et al. [18]	Cross sectional	2019	20 health regions of Saudi Arabia	Boys and girls, 6–14 y (NSBSP data)^a^	*N* = 44,259 (46.6% boys)	Interviewer administered questionnaire and clinical examinations	Overweight (85th to < 95th percentile), 6.4% (girls: 7.0% vs. boys: 5.8%); obesity (≥ 95th percentile), 4.1% (girls: 4.3% vs. boys: 3.8%); overweight and obesity, 10.5% (girls:11.3% vs. boys: 9.6%); eye refractory errors, 10.9% (girls: 12.1% vs. boys: 9.4%); dental caries, 38.7% (girls: 41.5% vs. boys: 35.6%)
Al-Dakheel et al. [22]	Cross sectional	2012	National	Boys and girls, 8–10 y (national goiter study)	*N* = 4,311 (52.2% boys)	Clinical examinations	Total goitre rate, 4.2% (girls:7.1%, boys:3.1%); 3.6% for grade 1 and 0.6% for grade 2; prevalence was < 5% in all parts of the country except the southern region, estimated at 12.7%
Al-Ghamdi et al. [23]	Cross sectional	2012	Riyadh, Jeddah, Tabuk, Dammam, Abha	Boys and girls, 15–19 y	*N* = 2,435 (54.6% boys)	Clinical examinations	Periodontitis, 8.6% (girls: 9.0% vs. boys: 8.2%)
Al-Ghamdi et al. [24]	Cross sectional	2012	Riyadh, Jeddah, Tabuk, Dammam, Abha	Boys and girls, 15–19 y	*N* = 2,435 (45.4% boys)	Clinical examinations	Slight gingivitis, 21.0% (girls: 17.7% vs. boys: 24.3%); moderate, 42.3% (girls: 41.2% vs. boys: 43.2%); severe, 1.8% (girls: 1.9% vs. boys: 1.8%)
Al-Hazzaa et al. [25]	Cross sectional	2019	Riyadh	Boys and girls, 6–12 y	*N* = 1,149 (45.5% boys)	Self-administered questionnaire, anthropometric data measured	Nocturnal sleep duration < 9 h, 65.8% (girls: 64.4% vs. boys: 67.5%)
Al-Hussein et al. [26]	Cross sectional	NA	Riyadh	Boys and girls, 6–17 y	*N* = 1,138 (53% boys)	Interview-administered questionnaire, anthropometric data measured, and blood samples collected	Elevated fasting blood sugar (≥ 6.1 mmol/l), 0.6% (girls: 0.9% vs. boys: 0.4%)
Al-Qahtani et al. [27]	Cross sectional	2014	Najran	Boys and girls, 7–19 y	*N* = 1700 (50.1% boys)	The Arabic version of the International Study of Asthma and Allergies in Childhood (ISAAC) questionnaire	Self-reported asthma, 27.5% (girls: 22.7% vs. boys 32.3%)
Baqal et al. [28]	Cross sectional	2011	13 regions of Saudi Arabia	Boys and girls, 10–18 y (Jeeluna study)	*N* = 12,463 (51.3% boys)	Self-administered questionnaire using the Youth Risk Behavior Survey and the Global School-based Student Health Survey; adolescents who engaged in PA for 1 or more d/wk were considered to “engage in PA”	Normal weight (5th to < 85th percentile), 54.8% (girls: 61.5% vs. boys: 48.8%); overweight (85th to < 95th percentile), 14.1% (girls: 14.5% vs. boys: 13.9%); obese (≥ 95th percentiles), 15.9% (girls: 11.0% vs. boys: 20.2%); underweight (< 5th percentile), 15.2% (girls: 13.0% vs. 17.2%); engage in PA, 53.4% (girls: 40.7% vs. boys: 68.3%); engage in PA (sports) at school, 35.4%; no of d spent on > 30 min exercise, 1.9 ± 2.4; television viewing (≥ 2 h/d), 42% (girls: 44.7% vs. boys: 40.4%);
Global Health Observatory data repository, WHO [29]	Cross sectional	2016	n.a.	Boys and girls, 5–19 y	—	—	Overweight (85th to < 95th percentile), 36.5%; obesity 18.0% (overall). Ages 10–19 y: overweight, 35.1%, obesity 16.7%. Ages 5–9 y: overweight, 36.5%; obesity 18.5%
Global Burden of Disease (GBD) data [3]	Cross sectional	2019	n.a.	Boys and girls, 5–14 y	—	—	Ages 5–14 y: total number of DALYs, 5,370 per 100,000 total population. Ages 15–19 y: mental health conditions, 19% of DALYs. Ages 5–14 y: mental disorders, 19.1% of DALYS
Household Health Survey [30]	Cross sectional	2018	13 regions of Saudi Arabia	Boys and girls, 15–19 y	*N* = 552,579	Interviewer administered Household Health Survey by the General Authority for Statistics (GASTAT) to each head of household	Dental health problems, 17.6% (girls: 18.7% vs. boys: 16.6%); diabetes, 2.9%; hypertension, 0.7%; current smoker, 2.1%, passive smokers (“second–hand” smokers), 25.8%; consumed the recommended intake of fruits and vegetables, 10.0%; sustained road traffic injuries, 14,559 adolescents; injured in other accidents (not road traffic related), 21,679 adolescents
Household Sports Practice Survey [31]	Cross sectional	2019	13 regions of Saudi Arabia	Boys and girls, 15–19 y	*N* = 26,000 (total)	Interviewer administered Household Sport Practice Survey by GASTAT to each head of household, questionnaires developed by PA experts on practicing sports activities	Practice sports activity (150 min and more per week), 23.8% (girls: 9% vs. boys: 38%)
Ibrahim et al. [32]	Cross sectional	2017	20 health regions of Saudi Arabia	Boys and girls, 10–19 y (National Obesity Survey)	*N* = 395,969 (43.7% boys)	Interviewer-administered questionnaire and anthropometric data measured	Underweight (BMI < 5th percentile), 28.4% (girls: 29.8% vs. boys: 27.2%); normal weight (5th to < 85th percentile), 53% (54% vs. 51.7%); overweight (85th to < 95th percentile), 11% (girls: 11.7% vs. boys: 10.3%); obese (≥ 95th percentile), 7.6% (girls: 7.1% vs. boys: 8.2%)
Moradi–Lakeh et al. [33]	Cross sectional	2013	13 regions of Saudi Arabia	Boys and girls, 15–19 y. (SHIS data)	*N* = 1,224 (58.6% boys)	Interviewer-administered questionnaire and blood samples collected. A short version of the International Physical Activity questionnaire was used to measure physical activity in occupational and recreational settings	Overweight, 20.9%; obesity 11.6%; normal blood pressure (SBP ≤ 120 and DBP ≤ 80); 64.4%, pre-hypertension (120 < SBP < 140 or 80 < DBP < 90); 31.3%, stage 1 hypertension (140 ≤ SBP < 160 or 90 ≤ DBP < 100), 3.9%; stage 2 hypertension (SBP ≥ 160 or DBP ≥ 100), 0.4%; current smoker, 3.5%; former smoker, 2.1%; never smoked, 94.4%; sheesha (water pipe) daily, 1.5%, other, 98.5%; no PA, 25.0% (girls: 42.8% vs. boys: 19.7%); insufficient PA, 29% (girls: 32.8% vs. boys: 22.1%); moderate PA, 12.6% (girls: 9% vs. boys 16%); vigorous PA, 33.0% (girls: 15.4% vs. boys 42.1%), sedentary behavior, 4.5 (SE = 0.1) hr (girls: 4.7 (0.1) vs. boys: 4.4 (0.1)); fruit and vegetable intake: no servings/d, 37%, 1 to 4 servings/d, 57.2%, ≥ 5 servings/d, 5.3%; always wore a seatbelt as a driver, 4.8%; never used a seatbelt as a driver, 60.6%; never used seatbelts as front passengers, 75.3%; always using a seatbelt, 2.8%; never used a seatbelt as back-seat passengers, 90.6%; always follow the speed limit 24.4%; never following the speed limit, 24.1%; using hands-free cellphones, 3.9%
Musharrafieh et al. [34]	Cross sectional	2011	13 regions of Saudi Arabia	Boys and girls, 10–18 y (Jeeluna study)	*N* = 11,348 (51.2% boys)	The Arabic version of the International Study of Asthma and Allergies in Childhood (ISAAC) questionnaire	Asthma, 8.2% (girls: 5.9% vs. boys 10.5%)
Nasim et al. [35]	Cross sectional	2011	13 regions of Saudi Arabia	Boys and girls, 10–12 y (Jeeluna study)	*N* = 12,121 (51% boys)	Self-administered questionnaire using the Youth Risk Behavior Survey and the Global School-based Student Health Survey; anthropometric data measured; blood samples collected	Sleep deprivation (< 7 h) on weekdays, 45.7%; on weekends, 33.5%
Shewear-AlAbdulrhman et al. [17]	Retrospective	2018	20 health regions of Saudi Arabia	Boys and girls, 6–16 y (NSBSP data)^a^	*N* = 12,032 (46.6% boys)	Interviewer-administered questionnaire and clinical examinations	Underweight (BMI < 5th percentile), 29.8% (girls: 30.8% vs. boys: 28.8%); normal weight (5th to < 85th percentile), 51.7% (girls: 53.3% vs. boys: 49.8%); overweight (85th to < 95th percentile), 9.7% (girls: 9% vs. boys: 10.4%); obese (≥ 95th percentile), 8.8% (girls: 6.9% vs. boys: 11.0%); low visual acuity (above 75th percentile), 15.3% (girls: 18% vs. boys: 12.3%); dental caries, 62.8% (girls: 57.9% vs. boys: 68.5%)
Saudi Health Interview Survey (SHIS) [36]	Cross sectional	2013	13 regions of Saudi Arabia	Boys and girls, ≥ 15 y	*N* = 1,224 (58.6% boys)	Interviewer-administered questionnaire, clinical examinations, and anthropometric measurements	Self-reported asthma, 3.4%; diabetes, 0.9% (girls: 0.9% vs. boys 0.8%)
Saudi Mental Health Survey (SNMHS) [37]	Cross sectional	2010	13 regions of Saudi Arabia	Boys and girls, 15–19 y	*N* = 317 (51% boys)	Interviewer-administered questionnaire; the Composite International Diagnostic Interview, a fully structured diagnostic interview designed to to assess common mental disorders and important correlates of these disorders in the general population	Lifetime mental disorder: any anxiety disorders, 23.3%; any mood disorder, 8.4%; any impulse disorders, 15.4%; any substance disorders, 3.2%; any eating disorders, 7.5%; any disorder, 40.4%

*BMI* Body mass index, *DBP* Diastolic blood pressure, *NSBSP* National School-Based Screening Program, *PA* Physical activity, *SHIS* Saudi Health Information Survey, *SBP* Systolic blood pressure, *WHO* World Health Organization

13 regions of Saudi Arabia (Al Riyadh, Makkah, Eastern Region, Northern Borders, Madinah, Jezan, Aseer, Najran, Qaseem, Tabuk, Hail, Al-Jouf, Al‐Baha). 20 educational regions (Al Riyadh, Jeddah, Eastern Province, Makkah, Al-Madinah, Al-Qassim, Al-Taif, Al-Qurayyat, Al-Jouf, Tabouk, Najran, Jazan, Hail, Al-Bahaa, Aseer, Al-Ahsa, Al- Qunfudhah, Hafar Al-Batin, Bisha, and the Northern Borders)

a. Also known as the Periodic Examination Program for School Students (PEPSS). n.a. = not applicable. — = not available

A total of 7 health conditions and 5 risk factors were identified. TableS2 provides an overview of publications presented by health conditions: (I) overweight and obesity; (II) mental health; IV) eye health; V) dental health; VI) asthma; VII) elevated blood glucose; VIII) high blood pressure; and behavioral risk factors: (I) tobacco use; (II) physical activity and sedentary behavior; IV) dietary patterns; V) sleep habits; VI) road traffic safety.

Description of global databases and national surveys used in the identified publications is presented in Table 2.. These include the following datasets: the Global Burden of Disease (GBD) data [3], the Household Sports Practice Survey [31], the National School-based Screening Program (NSBSP) [17, 18], the Household Health Survey [30], the National Obesity Survey [32], the Global Health Observatory (GHO) data repository/WHO [29], the Saudi National Mental Health Survey (SNMHS) [38], Saudi Health Interview Survey (SHIS) [36], the National survey on goiter prevalence [22], the Jeeluna Survey [20–35].

**Table 2.**
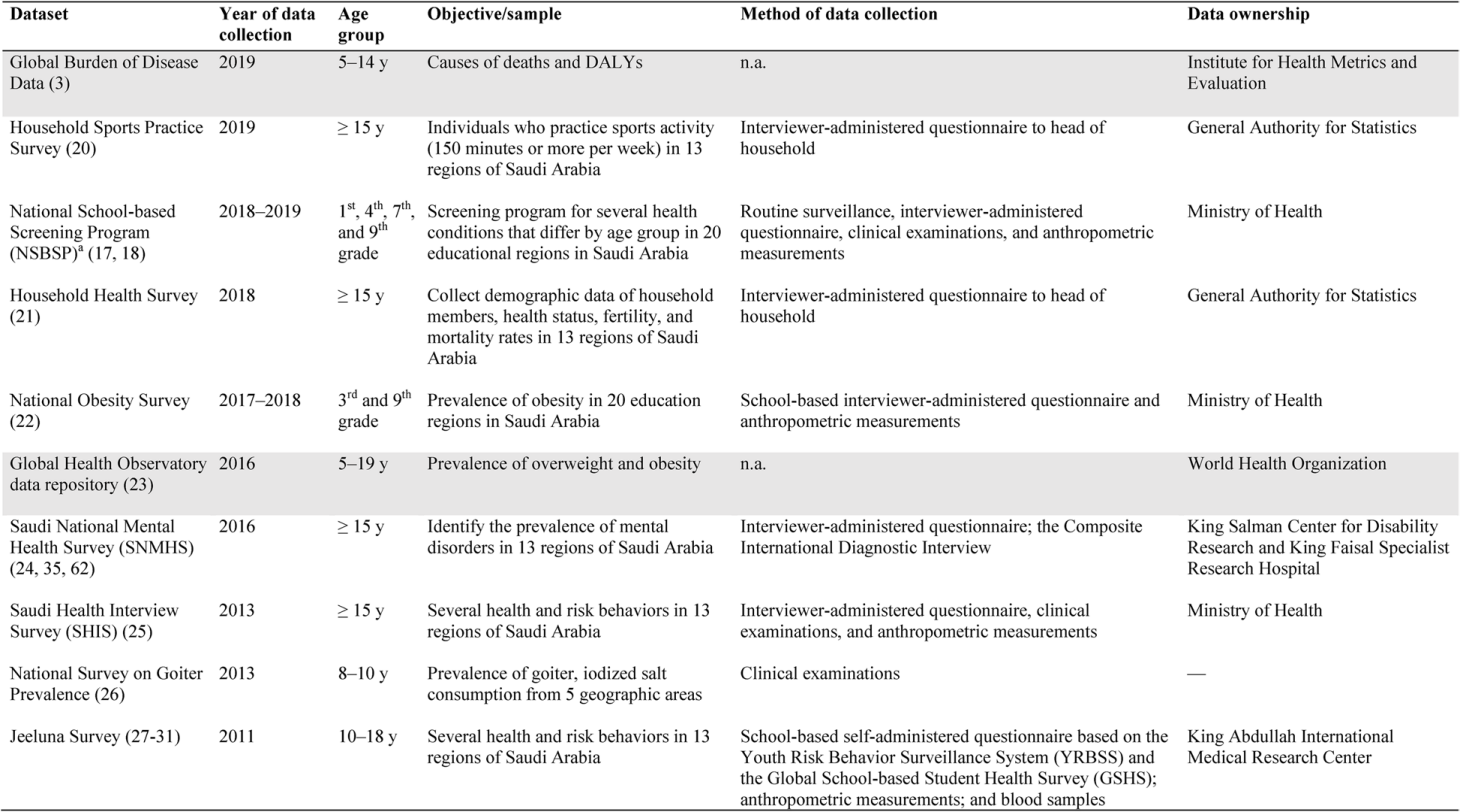
Description of datasets used by identified publications

Gray shading highlights international datasets. Disability-adjusted life years (DALYs). a. Also known as the Periodic Examination Program for School Students (PEPSS). n.a. = not applicable. — = not available

### Risk of bias assessment

Out of the studies assessed, six studies were classified as having a low risk of bias (scores: 7–8), six studies had a moderate risk of bias (scores: 5–6), and one seven was classified as having a high risk of bias (score: 4) (Table S3). Some studies demonstrated good representativeness of the sample and justification of sample size, though non-response bias was not addressed in some studies. Exposure measurement varied, with some studies relying on self-reported data (higher risk of bias) while others utilized validated clinical or objective measures (lower risk of bias). Studies that did not account for confounders in their analysis received lower comparability scores. Among the studies with a low risk of bias, Al-Buhairan et al. [21], Al-Dakheel et al. [22], and Al-Ghamdi et al. [23] scored 7–8 points, demonstrating strong methodology, appropriate statistical analysis, and adequate confounder adjustment. In contrast, Baqal et al. [28] scored 4 points, indicating a high risk of bias, primarily due to the lack of outcome validation and confounder control. Some studies used the same dataset but received different scores due to variations in statistical methods and the type of exposure/outcome measured (self-reported vs. objective measures).

### Publication bias

Funnel plots for outcomes with at least three studies are shown in (Figures S2 to S6). Visual inspection did not suggest marked asymmetry for overweight or obesity. Egger’s tests (Table S4) were not statistically significant for most outcomes. For example, overweight: intercept − 1.87, 95% CI − 41.47 to 37.74, *p* = 0.89; obesity: intercept 5.37, 95% CI − 46.35 to 57.09, *p* = 0.76. Estimates were also non-significant for mental health, dental health, asthma, elevated blood glucose, high blood pressure, tobacco use, physical activity, sleep, and road traffic safety (*p* > 0.05). Dietary indicators showed a borderline Egger result, intercept 61.79, 95% CI − 0.64 to 124.22, *p* = 0.052. Given the small number of studies for several outcomes, these tests have low power and should be interpreted with caution.

### Overweight and obesity

Included publications classified children and adolescents according to their body mass index (BMI) for age as follows: underweight (< 5th percentile); normal weight (5th to < 85th percentile); overweight (85th to < 95th percentile); obese (≥ 95th percentile) [39]. Anthropometric data in the included studies were measured by research team members.

Based on the 2016 GHO/WHO estimates, the overall prevalence of overweight and obesity in Saudi children and adolescents was 37% and 17%, respectively [29] (Table 1 and Table S2). Among those ages 10–19 years, the prevalence of overweight was 35% and obesity 17%; similarly, among the youngest age group of 5–9 years, the prevalence of overweight was 37% and obesity 19% [29]. In every age group, prevalence of overweight and obesity was higher among boys than girls (Figures S7 and S8).

The 2018–2019 NSBSP estimated overall prevalence of overweight at 6% and obesity at 4% [18]. Among a subgroup of only 1 st and 4th graders, the prevalence of overweight in children and adolescents was estimated at 10%, and obesity at 9% [17]. The 2017–2018 National Obesity Survey among 3rd and 9th graders estimated the prevalence of overweight at 11% (girls 12%, boys 10%), and of obesity at 8% (boys 8% and girls 7%) [32]. Despite the large sample size in these school-based surveys, it is unclear whether they are nationally representative of school-age children and adolescents. The 2013 SHIS reported that among those 15–19 years, the prevalence of overweight was 21% and obesity 12% [33]. The 2011 Jeeluna Survey for those 10–18 years reported the prevalence of overweight was 14% and obesity was 16% [28] (Table 1 and Table S2).

### Mental health

According to unpublished raw data obtained from the 2016 SNMHS [40], 40% of youth in Saudi Arabia had a mental health disorder at some point in their lifetime and 21% in the previous 12 months. The majority (boys and girls) were low-income (59%), and over half (55%) had a higher than average education with the vast majority living in urban areas (90%). Regional variations are in Figure S9 [40]. In 10–19 year old children, there was a 14% prevalence of overall mental health disorders [40]. Among adolescents with a prevalence of mental disorders at some point in their lifetime, the majority were boys—62% vs. 38% girls. The most frequent type of disorder was anxiety at 23% (Table 1 and Table S2).

The prevalence of mental health conditions among those younger than 10 years in Saudi Arabia is unknown. For those ages 10–18 years, the 2011 Jeeluna Survey reported that 14% suffered from sadness/depression, while 7% had anxiety [20]. This was measured using a self-administered Youth Risk Behavior Survey [41] guided by the Diagnostic and Statistical Manual of Mental Disorders criteria. The 2016 SNMHS used the Composite International Diagnostic Interview [42], a fully structured diagnostic interview designed to assess common mental disorders (anxiety, mood disorder, impulse disorder, eating disorder, and substance disorder) (Table 1 and Table S2).

### Eye health

The overall prevalence of refractory errors among school-age children and adolescents ranged between 11%–15%^1^ [17, 18]. Based on the NSBSP, among children and adolescents in all four grade categories included, the overall prevalence of refractory errors (myopia), termed “low visual acuity” in the study, was 15%^1^—girls 18% vs. boys 12% [17]. Among a subgroup of primary school students only (1st and 4th grade), only 11% had refractory eye errors [18]. Refractory errors are part of the NSBSP and are assessed by health professionals using Snellen charts, where students who scored 6 out of 12 were considered myopic [17, 18] (Table 1 and Table S2).

### Dental health

Based on the NSBSP, among children and adolescents in all four grade categories included, the overall prevalence of dental caries was 63% with higher rates among boys than girls—69% vs. 58% [17]. Among a subgroup of primary school students only, 39% had dental carries, with higher rates among girls than boys [18]. Oral health is part of the NSBSP and is assessed by dental professionals [17, 18].

The 2018 Household Health Survey showed that self-reported oral and dental health problems were prevalent in 18% of adolescents ages 15–19 years, and higher among girls (19%) than boys (17%) [30]. A third survey—a national survey among high-school students assessing the prevalence or periodontitis—found that 9% of older adolescents (ages 15–19 years) had periodontitis [23], 21% had slight gingivitis, 42% had moderate gingivitis, and 2% had severe gingivitis [24] (Table 1 and Table S2).

### Asthma

For those ages 10–18 years, the 2011 Jeeluna Survey found a 8% self-reported prevalence, with higher rates among boys than girls (11% vs. 6%) [34]. Based on a secondary analysis of the 2013 SHIS for those ages 15–19 years, there was a 3% self-reported prevalence of asthma [33]. However, for those ages 7–19 years, a 2021 study in Najran region found a 28% self-reported prevalence of asthma [27] (Table 1 and Table S2).

### Elevated blood glucose

The 2011 Jeeluna Survey for those ages 10–18 years estimated diabetes at around 0.7%, less prominent among boys than girls (0.6% vs. 0.9%) [21]. The 2013 SHIS for those ages 15–19 years estimated the prevalence was 0.9% (0.8% in boys and 0.9% in girls) [36]. The 2018 Household Survey for the same age group showed that 3% of adolescents self-reported diabetes [30]. Similarly, using blood samples from children and adolescents ages 6–17 years, the share of those with elevated fasting blood sugar (≥ 6.1 mmol/L) was 0.6% (girls 0.9%, boys 0.4%) [26] (Table 1 and Table S2).

### High blood pressure

The 2013 SHIS [33], using blood pressure measurements, reported that while the majority of adolescents ages 15–19 years in Saudi Arabia had normal blood pressure (64%), about 31% had pre-hypertension, 4% had stage 1 hypertension, and 0.4% had stage 2 hypertension. 2The 2018 Household Health Survey estimated that only 0.7% of adolescents had hypertension, based on self-reporting [30] (Table 1 and Table S2).

## Behavioral risk factors

### Tobacco use

The 2011 Jeeluna Survey reported that, among those ages 10–18 years, 16% had previously smoked cigarettes, while 11% had smoked *sheesha* (a water pipe) [21]. Disaggregated by gender, all types of smoking were more prevalent among boys than girls (22% vs. 10% for cigarette smoking and 14% vs. 7% for *sheesha*) [21]. The Jeeluna data were collected using a self-administered questionnaire that inquired about the frequency and type of smoking [21]. The 2013 SHIS for those 15–19 years old showed that the majority of adolescents never smoked (94%). Only 4% were cigarette smokers and 2% were former smokers and used *sheesha* daily [33]. The 2018 Household Health Survey, covering ages 15–19 years, reported a prevalence rate of 2% current smokers and 26% passive smokers (“second-hand” smokers) [30] (Table 1 and Table S2).

### Physical activity and sedentary behavior

The 2011 Jeeluna Survey estimated that among those ages 10–18 years, only 53% were “engaged in physical activity”, defined as performing physical activity for at least one day per week for 30 min or more each day; with boys more engaged in physical activity than girls (68% vs. 41%) [28]. The 2013 SHIS [33] showed that among ages 15–19 years, for physical activity, 25% reported none, 29% insufficient, 13% moderate, and 33% vigorous. The more recent 2019 Household Sports Practice Survey for those ages 15–19 years revealed that only 24% of adolescents ages 15–19 years spent 150 min or more per week in sports activities, more boys than girls (38% vs. 9%) [31]. Data for children under age 10 were not available.

Sedentary behavior was also prevalent among those ages 10–19 years. Per the 2011 Jeeluna Survey, about 42% of girls and boys spent at least 2 h per day watching television [28]. According to the 2013 SHIS, an average of 4.5 h per day were spent in sedentary behavior [33] (Table 1 and Table S2).

### Dietary habits and nutrient deficiencies

The 2011 Jeeluna Survey [21], for those ages 10–18 years, reported only 38% having more than one serving a day of fruits and 54% of vegetables. The 2013 SHIS [33] reported that among those 15–19 years old, only 5% had the recommended five servings or more a day of fruits and vegetables, and 37% had no servings of fruits and vegetables. The 2018 Household Health Survey among those ages 15–19 years reported that 10% consumed the recommended intake of fruits and vegetables [30]. All three surveys used self-reporting measures; the 2013 SHIS used a food frequency questionnaire [33].

Per the Jeeluna Survey, among those ages 10–18 years, 38% had two or more drinks per day of carbonated beverages (boys 44%, girls 30%), and 22% had more than one serving a day of energy drinks (boys 26%, girls 18%) [21].

For vitamin D status, the 2011 Jeeluna Survey used a blood sample analysis and found 96% of all adolescents were vitamin D deficient (defined as less than 50 nmol/L) [21]. Goiter prevalence, caused by long-term depletion of iodine storage leading to serious neurological disorders [43], was low (4%) in Saudi Arabian children overall in 2013 but high at 13% in the southern region, where a high proportion of households were not consuming enough iodized salt [22] (Table 1 and Table S2).

### Sleep habits

The 2011 Jeeluna Survey for those 10–18 years reported that 46% had sleep deprivation (sleeping less than 7 h a day) on weekdays [35]. About 66% of younger children and adolescents had less than 9 h of sleep ^(42)^ (Table 1 and Table S2).

### Road traffic safety

The 2011 Jeeluna Survey of adolescents ages 10–18 years reported that only 14% sometimes/always wore seatbelts and 18% would drive a car without permission [21]. The 2013 SHIS [33] showed that 75% of adolescents ages 15–19 years never used seatbelts as front passengers and only 3% reported always using a seatbelt. As back-seat passengers, 91% never wore seatbelts. More alarmingly, only 5% of drivers always wore a seatbelt, with 61% reporting never using a seatbelt as a driver.

The 2013 SHIS reported than only 24% of adolescents always follow the speed limit and 24% reporting never following the speed limit. Additionally, only 4% reported always using hands-free cellphones while driving. All of these were self-reported [33] (Table 1 and Table S2).

## Discussion

The present review presents the risk factors and health conditions contributing to morbidity and mortality in school-age children and adolescents in Saudi Arabia. Several health concerns were identified, including overweight and obesity, mental health, eye health, dental health, and chronic conditions. Several behavioral factors were also included, such as tobacco use, physical activity and sedentary habits, dietary patterns, sleep habits, and road traffic safety.

In Saudi Arabia, the estimated prevalence of overweight in children and young adolescents as per the 2018–2019 NSBSP data (6–16 years) [17, 18] showed varying estimates, ranging between 6% and 10%. Obesity estimates ranged from 16% in 2011, 12% in 2013, to 4% in 2019 [18, 28]. These figures are significantly lower than the estimated overweight and obesity rates reported in GHO/WHO [29] (35% and 17%, respectively), but are higher than global data, with the most recent global obesity estimates in 2016 by the Non-communicable Disease Risk Factor Collaboration Study [44] reporting obesity prevalence of 8% in boys and 6% in girls aged 5–18 years. Meanwhile, the 2013 prevalence of overweight and obesity in children and adolescents (5–18 years) in developing countries reached 13% in boys and 13% in girls [45], comparable to local estimates of overweight status for the same age group, which was higher in girls (12%) than boys (10%) [32].

Mental health conditions were the leading contributor to disease burden and disability among adolescents in Saudi Arabia [1, 3]. These include conditions such as anxiety, depression, and behavioral disorders. Mental health conditions accounted for 19% of disability adjusted life years (DALYs) among those ages 15–19 years and were the leading risk factor driving morbidity among this age group [3]. About 19% of total DALYS among those ages 5–14 years were caused by mental disorders [3]. In the Jeeluna study, about 14% of adolescents aged 10–12 years reported sadness/depression and 7% reported anxiety [20]. The 2016 SNMHS [40] showed that in older adolescents aged 15–19 years, the prevalence of any anxiety and any mood disorder were much higher than global prevalences of 4% in children aged 3–17 diagnosed with depression and 9% of children aged 3–17 years with anxiety in 2016–2019 [46]. It is worth noting that reports in Saudi Arabia were all based on self-administered questionnaires, while global data were based on clinical diagnosis. Further, while the 2016 Jeeluna prevalence rates might appear lower than the 2016 SNMHS, the results are incomparable due to different data collection years, age groups included, and tools applied.

In the present review, we focused on eye refractory errors (e.g., myopia, also known as nearsightedness, the most common eye disorder among children and adolescents) which can undermine children’s and adolescents’ academic performance if left untreated [47]. Eye refractory errors and low visual acuity in Saudi children and adolescents reported from the NSBSP data ranged between 11% and 15% [17, 18], comparable to or even higher than global estimates of 12% [48].

The estimated prevalence of dental caries reported from NSBSP data [17, 18] were 39% and 63%, respectively. Both values were assessed through dental exams and were lower than the 2020 global prevalence of about 54%, which was considered high [49]. The prevalence of dental caries in Saudi children and adolescents was also lower than estimates from the Eastern Mediterranean Region of 61% in adolescents aged 12 years, 70% in those aged 15 years, and 66% in those aged 6–15 years [50]. Self-reported oral and dental health problems from the 2018 Household Health Survey showed lower figures of 18% in adolescents ages 15–19 years [30]. Other surveys reported the prevalence or periodontitis was 9% [23], slight gingivitis 21%, moderate gingivitis 42% had, and severe gingivitis 2% [24]. The results from the three surveys cannot, however, be directly compared because one was self-reported and the other two had different clinical definitions of dental health.

The prevalence of asthma is not routinely measured in the NSBSP. Secondary analysis of the 2013 SHIS showed a 3% prevalence of asthma [33], a higher estimate from the Jeeluna study (8%) [34], and an even higher prevalence (28%) from a study in Najran in younger children and adolescents aged 7–19 years [27], all using self-reported data. Worldwide asthma prevalence used different age-groups and represented 10% in 13–14-year olds and 10% in 6–7-year olds [51], with asthma symptoms reported from asthma centers.

Data from the last decade or so suggest that the prevalence of diabetes is generally low among those ages 10–19 years in Saudi Arabia, at 1–3% [21, 30, 33]. Generally, the diagnosis of type 1 and 2 diabetes in children and adolescents considers the presence of clinical features, for example, polydipsia and polyuria, in addition to hyperglycemia, for example, random plasma glucose of ≥ 11.1 mmol/L [52]. Globally, type 2 diabetes prevalence per 1000 children and adolescents aged 10 to 19 years was estimated at 0.67% in 2017, increasing significantly from 0.34% in 2001 [53]. The type of diabetes was not specified in the identified studies of this review, however, and was self-reported [21, 30, 33], making comparisons impossible.

Elevated blood pressure in children and adolescents is defined as having a systolic or diastolic blood pressure above the 95th percentile for sex, age, and height [54]. The 2018 Household Health Survey [30] reported that 0.7% of older adolescents aged 15–19 years were hypertensive, while the 2013 SHIS data showed a higher prevalence (4%) of stage 1 hypertension in the same age group [30]. Both estimates are lower than estimates mainly for adolescents in the United States showing a pre-hypertension prevalence range of 12–17% [55].

The prevalence rate of tobacco use varied across different data sources and measurement tools in Saudi Arabia over the last decade or so. In the Jeeluna study, 22% of adolescent boys and 10% of girls had previously smoked cigarettes [21], while the 2013 SHIS data, using another self-administered questionnaire with varying criteria, reported a lower prevalence of 4% currently smoking cigarettes and 2% former smokers [33]. The 2018 Household Health Survey reported a prevalence of 2% current smokers and 26% passive smokers [30]. The three surveys used self-reporting but had different questionnaires, making them impossible to compare to each other or to global data. Globally, the prevalence of cigarette smoking was 11% in boys and 6% in girls ages 13–15 years in 2018, based on cigarette smoking of at least 1 day during the past 30 days [56].

Most Saudi adolescents do not meet the WHO recommended guidelines [57] for optimum physical activity and all reports presented in this review used different tools to measure physical activity. Specifically, the 2011 Jeeluna Survey identified physical activity using a self-administered questionnaire based on the Youth Risk Behavior Surveillance System and the Global School-based Student Health Survey [28], the 2013 SHIS [33] used a short version of the International Physical Activity Questionnaire [58] among adolescents ages 15–19 years, categorizing participants as insufficiently active, sufficiently active, and highly active, and the 2019 Household Sports Practice Survey asked individuals to self-report on their practicing of sports [31].

Saudi children and adolescents’ intake of fruit and vegetables does not meet global [59] and national [60] recommendations, with only 38% having ≥ 1 serving/day of fruit while 54% (≥ 1 serving/day) of vegetable [21] and 37% of no servings per day [33]. For nutrient status, vitamin D, an essential nutrient for bone health where deficiency can increase respiratory disease risk and lead to adverse chronic conditions in adulthood [61], yet most Saudi Arabian children and adolescents ages 10–19 years have shown vitamin D deficiency but normal levels of iodine.

In Saudi adolescents, sleep hours were lower than the recommended amount by the American Academy of Sleep Medicine [62], in which children 6 to 12 years of age should sleep 9 to 12 h and older children 13 to 18 years of age should sleep 8 to 10 h for optimal health. Noteworthy is that sleep hours were all identified using self-reported questionnaires [25, 35].

The leading cause of mortality among children and adolescents aged 5–14 years in 2019 was road traffic injuries at 25% of total deaths [3]. In an older age group of 15–19 years, the 2018 Household Health Survey showed 14,559 adolescents self-reporting that they had sustained road traffic injuries and 21,679 were injured in other accidents (not road traffic related) [30]. The majority of Saudi Arabian adolescents do wear seatbelts, use hands-free cellphone devices while driving, nor do they follow the recommended speed limits [33].

Results of this systematic review can be used to form a set of recommendations of priority health conditions and behavioral risk factors. For example, interventions to enhance road and traffic safety should be implemented (e.g., increasing seatbelt use), especially considering traffic injuries have been a leading cause of death in this age group in Saudi Arabia since 1990 and are a major contributor to death and DALYs [3]. Additionally, other leading causes of DALYs should be addressed in interventions (i.e., mental disorders, nutritional deficiencies, diabetes, substance use).

The present review has several limitations. One of the key challenges is the heterogeneity in methodologies across different sources reporting the prevalence of health conditions and risk factors. The risk of bias assessment revealed variations in study quality, with methodological differences impacting the reliability of prevalence estimates. Differences in sample representativeness, data collection periods, and study settings (e.g., national surveys vs. regional studies) may have further influenced the reported estimates. While the 12 health conditions and risk factors chosen for this study were based on those commonly measured by the WHO [15] and CDC [16], there may be other factors contributing to the health and well-being of school-age children in Saudi Arabia that were not included. Furthermore, several large-scale studies were included in the review; however, the conclusions drawn from each are subject to the limitations inherent in the original work. For instance, information on several priority health conditions and behavioral risk factors was collected via self-report (e.g., diabetes and hypertension prevalence), introducing bias and making comparisons with clinical diagnoses difficult. This likely contributed to the wide range of prevalence estimates (e.g., overweight and obesity estimates ranging from 4% to 37%). National data were not necessarily weighted to the population, causing a wide range of estimates, and different studies used different measurement tools, making comparisons difficult (e.g., for physical activity). These methodological inconsistencies limit direct comparability across studies and should be carefully considered when interpreting findings. Higher quality and purposefully collected data are needed to make results comparable, representative, and less biased. Data on many health conditions and risks in this report come from the 2011 Jeeluna study and thus data that is at least a decade old. More recent surveillance can better inform current priorities and form baseline measures to quantify intervention successes. Finally, this study revealed several areas where the literature is scarce. Data are needed for almost all health conditions or risk factors for those under 15 years of age, particularly those aged 5 to 9. The morbidity for those 5 to 9 years is poorly documented and not systematically collected, and current national surveys only include individuals over 15 years of age.

## Conclusions

This review presents priority health conditions and behavioral risks among school-aged children and adolescents in Saudi Arabia. Several priority health conditions and behavioral risk factors examined are areas of concern for school-aged children and adolescents, either due to a higher prevalence compared to global or regional values, increasing rates over time, or not meeting recommendations and guidance. Addressing these concerns requires efforts to fill existing knowledge gaps by providing more comprehensive, higher-quality, and up-to-date information. Such data are essential for informing policy decisions and guiding the development of effective interventions. Effective school health strategies can be created with sufficient information and understanding of the existing health risks for children and adolescents. Future research should focus on standardized data collection methods, including the use of consistent diagnostic criteria, validated measurement tools, and representative samples to improve comparability and reliability of prevalence estimates. In addition, research should prioritize robust methodologies, well-defined sampling strategies, and appropriate confounder adjustments to further enhance data comparability and reliability.

## Supplementary Information

Supplementary Material 1.

Supplementary Material 2.

Supplementary Material 3.

## Data Availability

No datasets were generated or analysed during the current study.

## Abbreviations

BMI: Body Mass Index
DALY: Disability-adjusted Life Year
GBD: Global Burden of Disease
GHO: Global Health Observatory
NSBSP: National School-Based Screening Program
PA: Physical Activity
SHIS: Saudi Health Interview Survey
SNMHS: Saudi National Mental Health Survey
WHO: World Health Organization

## Footnotes

1 The numbers reported in the article text do not match those reported in the tables. We used the numbers in the text.

2 Normal blood pressure (BP) was defined as systolic blood pressure (SBP) ≤120 and diastolic blood pressure (DBP) ≤80 mmHg; prehypertension 120< SBP <140 or 80< DBP <90 mmHg; stage 1 hypertension 140≤ SBP <160 or 90≤ DBP <100 mmHg; and stage 2 hypertension SBP ≥160 or DBP ≥100 mmHg, based on the average of three measurements.
