# Supplementary material for: Health of school-age children and adolescents in Saudi Arabia: a systematic review
Source: BMC Public Health. 2026 Jan 26;26:644. doi: 10.1186/s12889-025-25897-x (PMC12915014; doi:10.1186/s12889-025-25897-x)
Supplement: Supplementary file 2 — Supplementary Material 2. [file 12889_2025_25897_MOESM2_ESM.docx]

FIGURE S1

Health conditions and risk factors typically addressed in school health–based programs

| Health conditions | Risk factors |
| --- | --- |
| Overweight and obesity | Tobacco use |
| Mental health | Physical activity and sedentary habits |
| Eye health | Dietary patterns |
| Dental health | Sleep habits |
| Chronic conditions^a^ | Road traffic safety |

*Note:* a. These include asthma, elevated blood glucose, and high blood pressure.

FIGURE S2

Overweight among Saudi children and adolescents, by sex and age group, WHO GHO 2016.


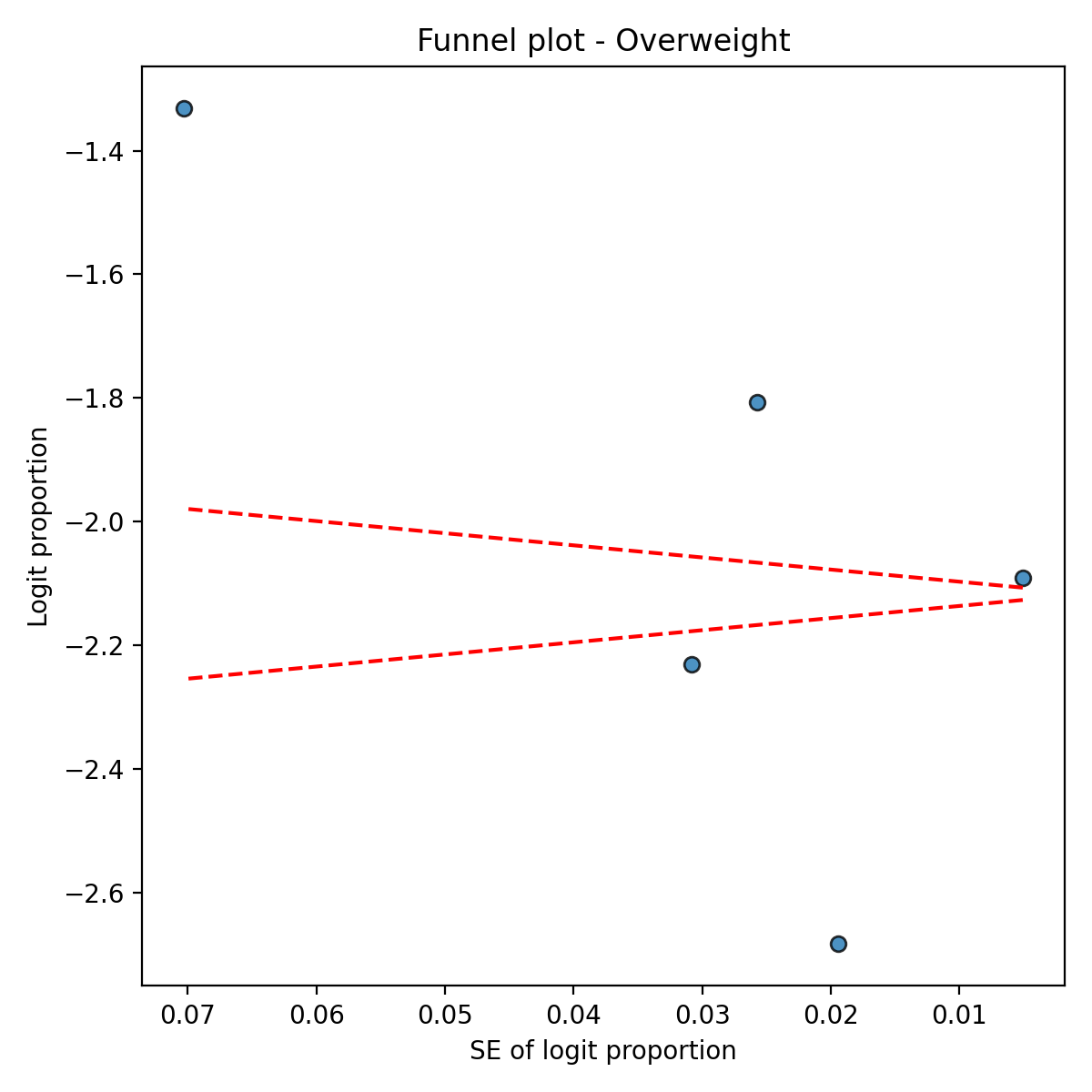


FIGURE S3

Obesity among Saudi children and adolescents, by sex and age group, WHO GHO 2016.


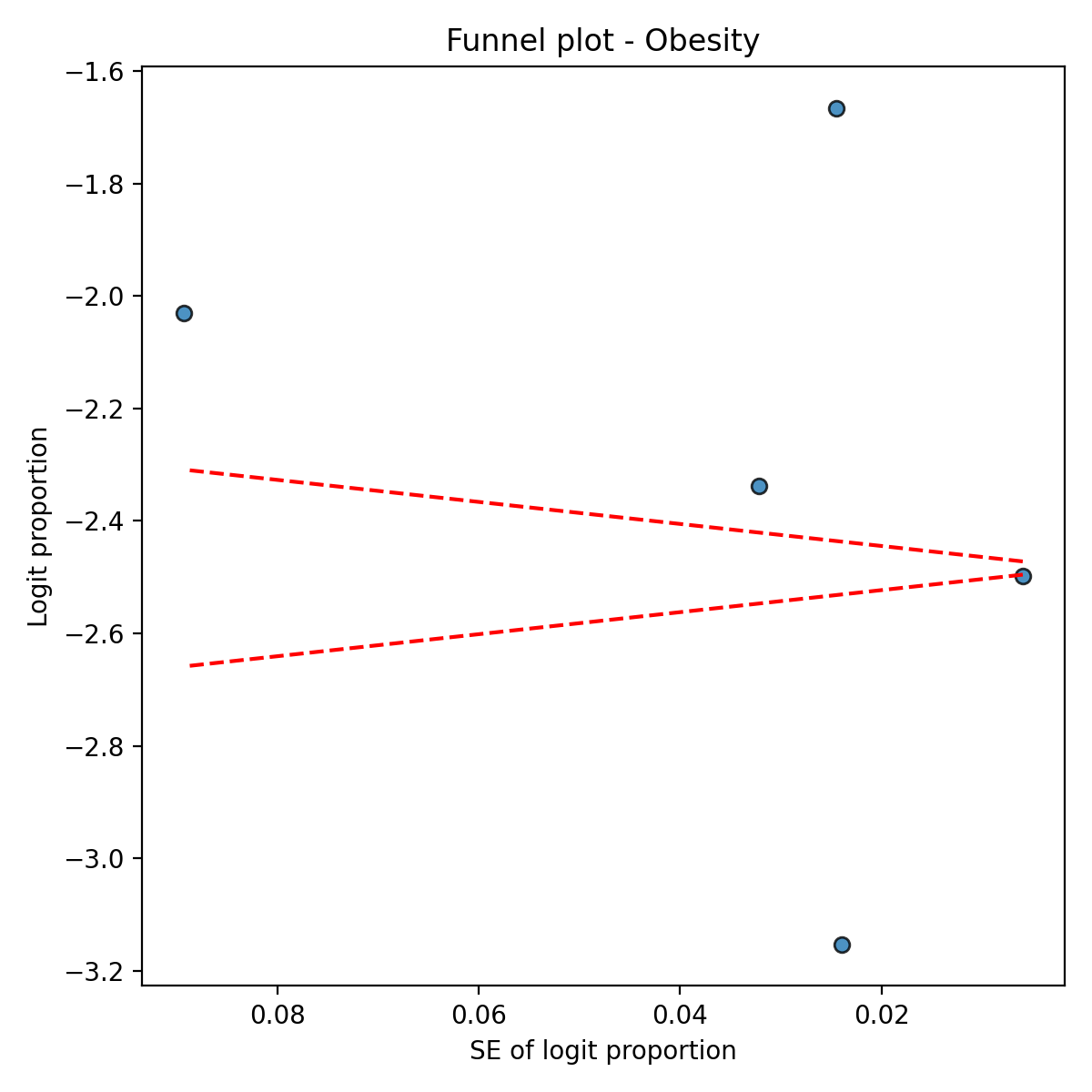


FIGURE S4

Regional prevalence of any mental health disorder among Saudi adolescents, SNMHS 2016.


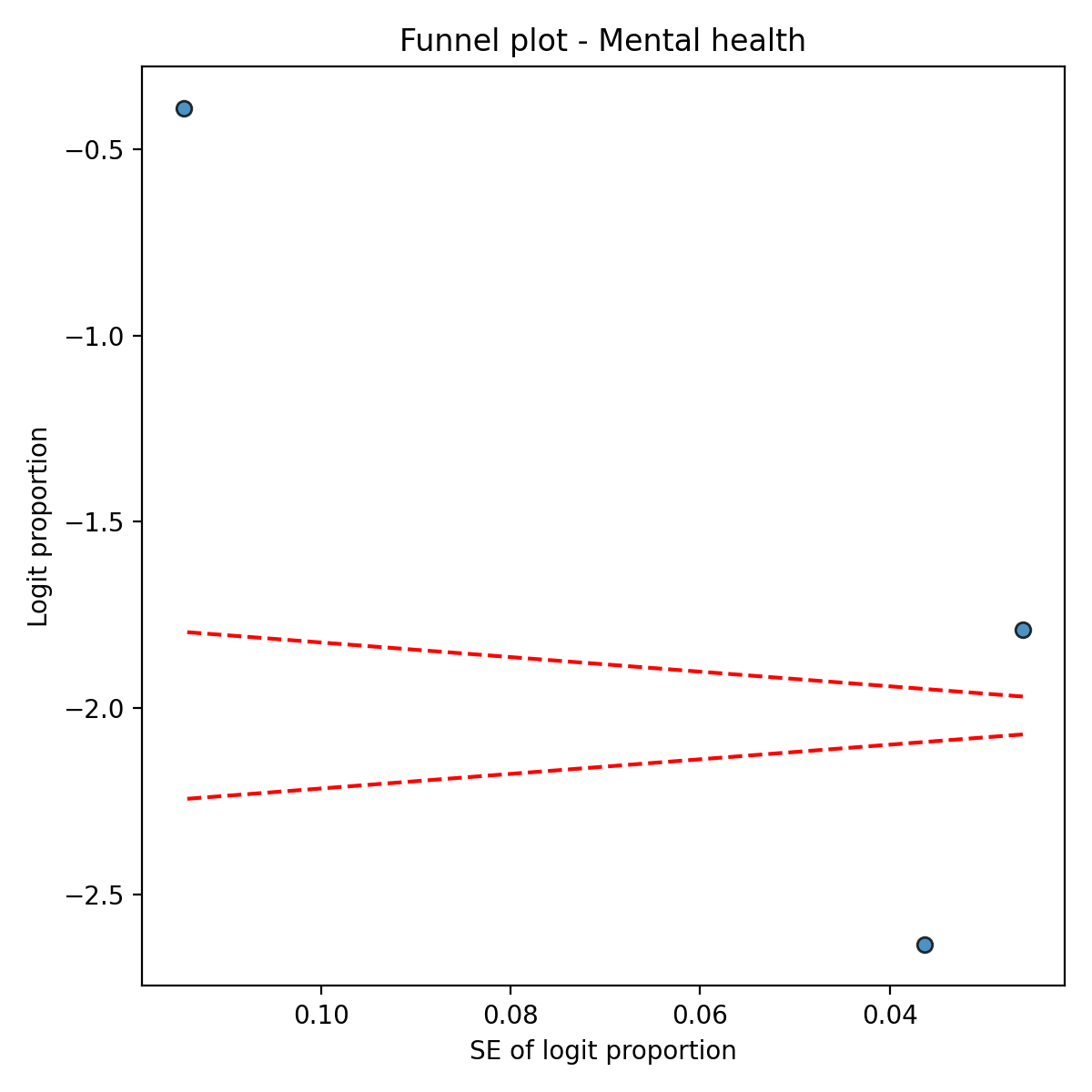


FIGURE S5

Funnel plot of included studies for overweight prevalence in Saudi children and adolescents.


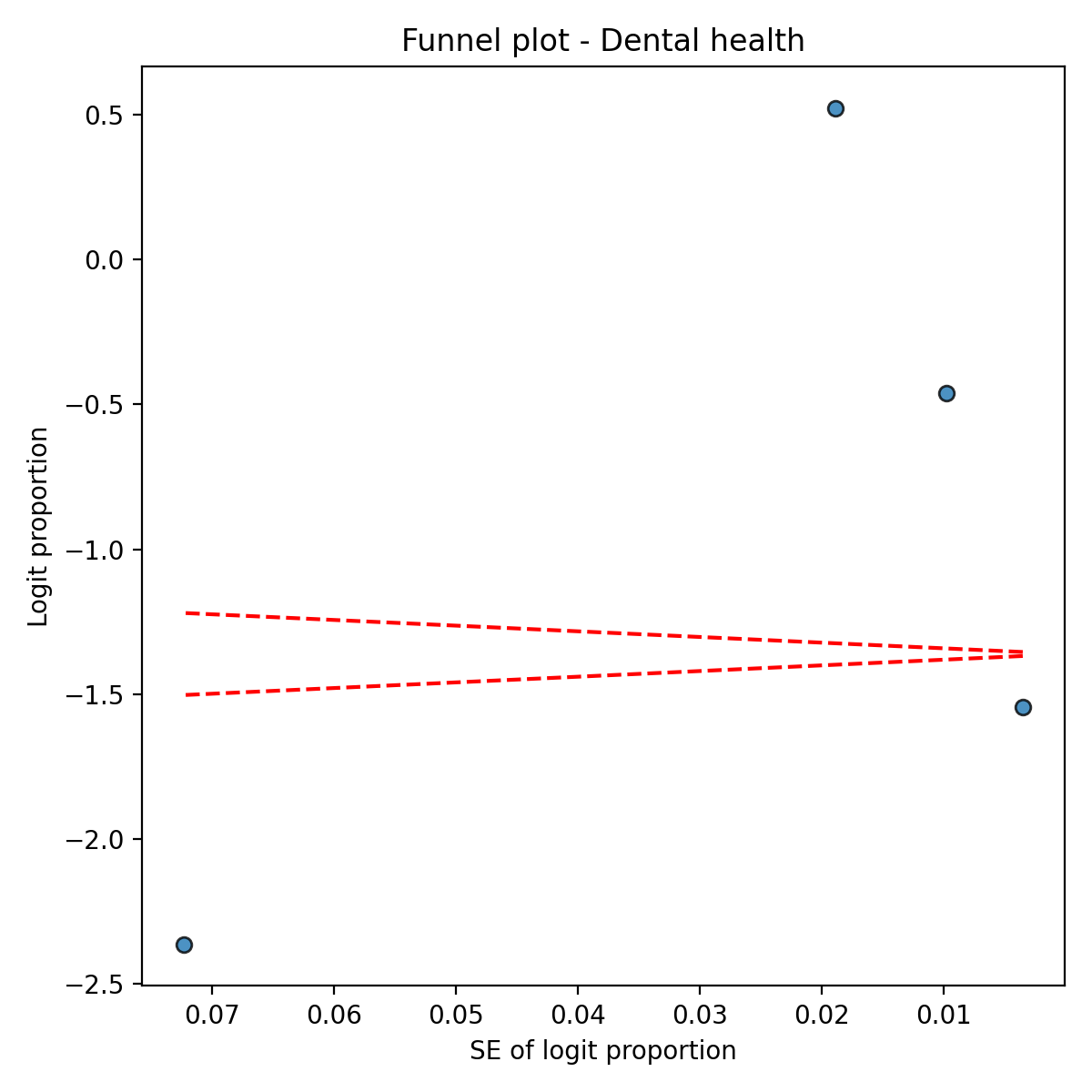


FIGURE S6

Funnel plot of included studies for obesity prevalence in Saudi children and adolescents.


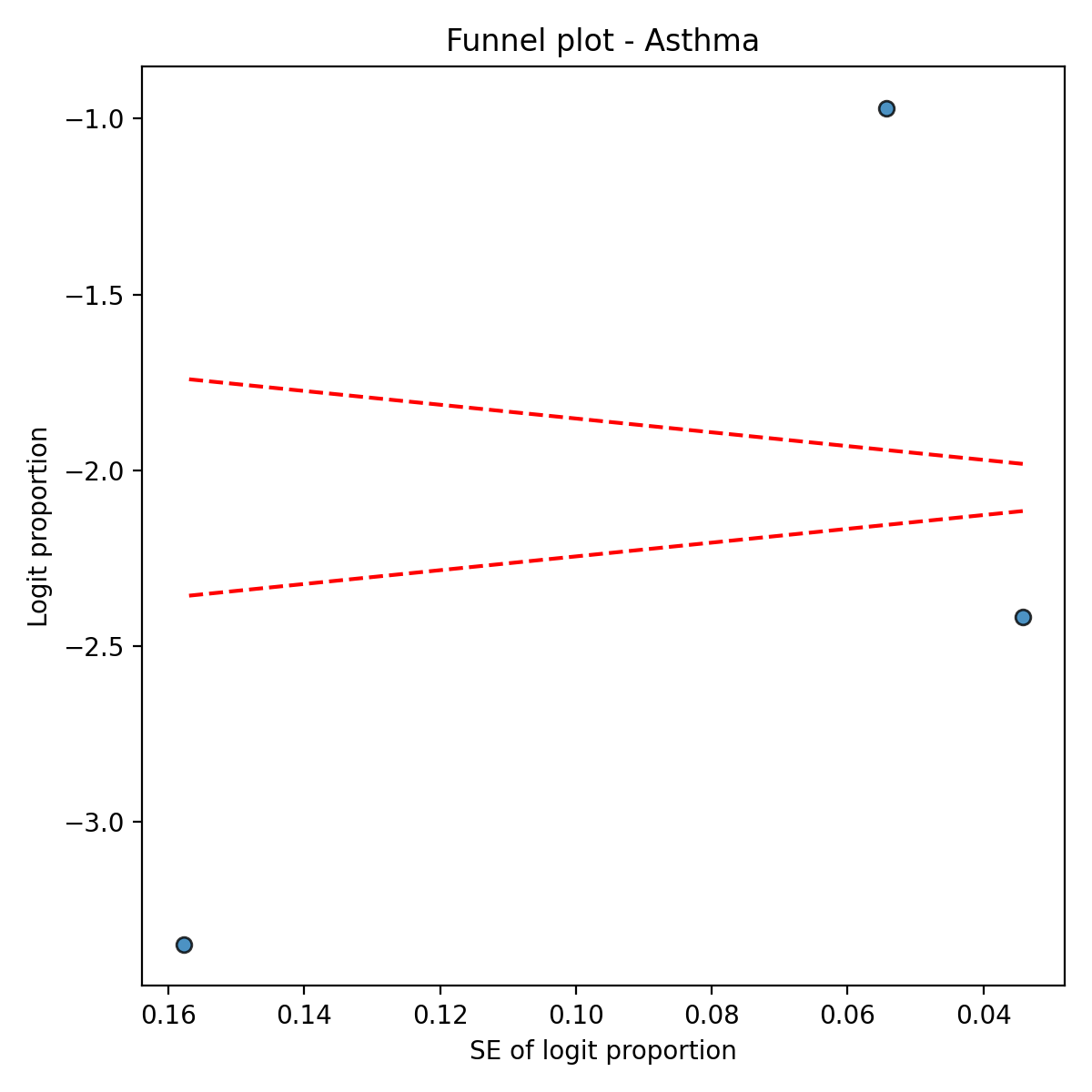


FIGURE S7

Prevalence of overweight among ages 5–19 years in Saudi Arabia by gender and age group

*Source:* WHO (World Health Organization). 2020. The Global Health Observatory (database). Geneva: WHO. https://www.who.int/data/gho.

*Note:* Overweight body mass index > +1 standard deviation above the median (crude estimate) (%).

FIGURE S8

Prevalence of obesity among ages 5–19 years in Saudi Arabia by gender and age group

*Source:* WHO (World Health Organization). 2020. The Global Health Observatory (database). Geneva: WHO. https://www.who.int/data/gho.

*Note:* Obesity body mass index > +2 standard deviations above the median (crude estimate) (%).

FIGURE S9

Regional distribution of adolescents with mental health disorders in Saudi Arabia, 2016

*Source:* Unpublished raw data from Altwaijri et al. 2020. “The Saudi National Mental Health Survey: Survey Instrument and Field Procedures. *Int J Methods Psychiatr Res*. 29:e1830. <https://doi.org/10.1002/mpr.1830>
