# Supplementary material for: Health of school-age children and adolescents in Saudi Arabia: a systematic review
Source: BMC Public Health. 2026 Jan 26;26:644. doi: 10.1186/s12889-025-25897-x (PMC12915014; doi:10.1186/s12889-025-25897-x)
Supplement: Supplementary file 3 — Supplementary Material 3. [file 12889_2025_25897_MOESM3_ESM.docx]

**Table S1. Search strategy for PubMed**

| “oral health”[mesh] OR “oral health”[tiab] OR “oral care”[tiab] OR “gingival”[tiab] OR “dental”[tiab] OR “mental health”[mesh] OR “mental health”[tiab] OR “social”[tiab] OR “emotional health”[tiab] OR “wellbeing”[tiab] OR “environment”[mesh] OR “environment”[tiab] OR “pollution”[tiab] OR “smoking”[mesh] Or “smoking”[tiab] OR “tobacco”[tiab] OR “sleep”[mesh] OR “sleep”[tiab] OR “nutritional sciences”[mesh] OR “child nutrition sciences”[mesh] OR “nutrition assessment”[mesh] OR “nutrition”[tiab] OR “nutrition education”[tiab] OR “diet”[mesh] OR “diet”[tiab] OR “snack”[tiab] OR "meal”[tiab] OR "lunchbox"[tiab] OR “dietary behavior”[tiab] OR “breakfast consumption”[tiab] OR  “exercise”[mesh] OR “exercise”[tiab] OR “physical activity”[tiab] OR “fitness”[tiab] OR “sport”[tiab] OR “movement”[tiab] OR “active”[tiab] OR “physical education”[tiab] OR “sedentary behavior”[mesh] OR “sedentary behavior”[tiab] OR “screen time”[tiab]  OR "curriculum"[mesh] OR "curriculum"[tiab] OR "education"[mesh] OR "education"[tiab]OR “obesity”[tiab] OR “obesity”[mesh] OR ”overweight”[tiab] OR “visual”[tiab] OR “visual impairment”[tiab] OR “vision”[tiab] OR “vision health” [tiab] OR “vision screening”[tiab] OR “eye health”[tiab] OR “asthma”[mesh] OR “asthma”[tiab] OR “asthmatic”[tiab] OR “morbidity”[tiab] OR “morbidity”[mesh] OR “mortality”[tiab] OR “mortality”[mesh] AND “school”[tiab] OR “school health”[tiab] OR “wellness”[tiab] OR “child”[mesh] OR “child”[tiab] OR “child health”[mesh] OR “child health”[tiab] OR “adolescent”[mesh] OR “adolescent”[tiab] OR “adolescent health”[tiab] OR “school children”[tiab] OR “schoolchildren”[tiab] OR “childhood”[tiab] OR “youth”[tiab] OR “students”[tiab] AND “Saudi Arabia”[tiab] OR “Saudi”[tiab] OR “Riyadh”[tiab] OR “Jeddah”[tiab] OR “Mecca”[tiab] OR “Dammam”[tiab] OR “Abha”[tiab] OR “Khobar”[tiab] OR “Taif”[tiab] OR “Tabuk”[tiab] OR “Aseer”[tiab] NOT “animal” |
| --- |

**Table S2. overview of publications presented by health conditions and risk factors**

| **Health issue/risk factor** | **Reference** | **Results** |
| --- | --- | --- |
| *Overweight and obesity* | Al-Daajani et al. [1] | Overweight (85th to <95th percentile), 6.40% (girls: 7.0% vs. boys: 5.8%); obese (≥95th percentile), 4.10% (girls: 4.3% vs. boys: 3.8%); overweight and obese, 10.50% (girls:11.3% vs. boys: 9.6%) |
|  | Shewear-AlAbdulrhman et al. [2] | Underweight (BMI<5th percentile), 29.8% (girls: 30.8% vs. boys: 28.8%); normal weight (5th to <85th percentile), 51.7% (girls: 53.3% vs. boys: 49.8%); overweight (85th to <95th percentile), 9.7% (girls: 9% vs. boys: 10.4%); obese (≥95th percentile), 8.8% (girls: 6.9% vs. boys: 11.0%) |
|  | Global Health Observatory data repository, WHO [3] | Overweight (85th to <95th percentile), 36.5%; obesity 18% (overall). Ages 10–19 y: overweight, 35.1%, obesity 16.7%. Ages 5–9 y: overweight, 36.5%; obesity 18.5% |
|  | Ibrahim et al. [4] | Underweight (BMI<5th percentile), 28.4% (girls: 29.8% vs. boys: 27.2%); normal weight (5th to <85th percentile), 53% (54% vs. 51.7%); overweight (85th to <95th percentile), 11.0% (girls: 11.7% vs. boys: 10.3%); obese (≥95th percentile), 7.6% (girls: 7.1% vs. boys: 8.2%) |
|  | Moradi-Lakeh et al. [5] | Overweight, 20.9%; obesity 11.6% |
|  | Baqal et al. [6] | Normal weight (5th to <85th percentile), 54.8% (girls: 61.5% vs. boys: 48.8%); overweight (85th to <95th percentile), 14.1% (girls: 14.5% vs. boys: 13.9%); obese (≥95th percentiles), 15.9% (girls: 11.0% vs. boys: 20.2%); underweight (<5th percentile), 15.2% (girls: 13.0% vs. 17.2%) |
| *Mental health* | Global Burden of Disease (GBD) data [7] | Ages 5–14 y: total number of DALYs, 5,370 per 100,000 total population. Ages 15–19 y: mental health conditions, 19% of DALYs. Ages 5–14 y: mental disorders, 19.1% of DALYS |
|  | Saudi Mental Health Survey (SNMHS) [8] | Lifetime mental disorder: Any anxiety disorders, 23.3%; any mood disorder, 8.4%; any impulse disorders, 15.4%; any substance disorders, 3.2%; any eating disorders, 7.5%; any disorder, 40.4% |
|  | AbouAbbas et al. [9] | Sadness/depression, 14.3% (girls: 19% vs. boys: 10.1%); anxiety, 6.7% (girls: 9.1% vs. boys: 4.6%); older adolescents (>15 y) reported feeling so sad or hopeless, 59%; worried, 66%; exposure to bullying at school during the preceding 30 d, 25.0% (girls: 22.7% vs. boys: 27.1%); involved in physical violence at school in the preceding year, 20.0% (girls: 11.7% vs. boys: 28.9%) |
| *Eye health* | Al-Daajani et al. [1] | Eye refractory errors, 10.90% (girls: 12.1% vs. boys: 9.4%) |
|  | Shewear-AlAbdulrhman et al. [2] | Low visual acuity, 15.3% (girls: 17.9% vs. boys: 12.3%) |
| *Dental health* | Shewear-AlAbdulrhman et al. [2] | Dental caries, 62.8% (girls: 57.9% vs. boys: 68.5%) |
|  | Al-Daajani et al. [1] | Dental caries, 38.7% (girls: 41.5% vs. boys: 35.6%) |
|  | Household Health Survey [10] | Dental health problems, 17.6% (girls: 18.7% vs. boys: 16.6%) |
|  | Al-Ghamdi et al. [11] | Periodontitis, 8.6% (girls: 9.0% vs. boys: 8.2%) |
|  | Al-Ghamdi et al. [12] | Slight gingivitis, 21% (girls: 17.7% vs. boys: 24.3%); moderate, 42.3% (girls: 41.2% vs. boys: 43.2%); severe, 1.8% (girls: 1.9% vs. boys: 1.8%) |
| *Asthma* | Saudi Health Interview Survey (SHIS) [13] | Self-reported asthma, 3.4% |
|  | Musharrafieh et al. [14] | Self-reported asthma, 8.2% (girls: 5.9% vs. boys 10.5%) |
|  | Al-Qahtani et al. [15] | Self-reported asthma, 27.5% (girls: 22.7% vs. boys 32.3%) |
| *Elevated blood glucose* | Household Health Survey [10] | Diabetes, 2.9% |
|  | Saudi Health Interview Survey (SHIS) [13] | Diabetes, 0.9% (girls: 0.9% vs. boys 0.8%) |
|  | Al-Buhairan et al. [16] | Diabetes, 0.7% (girls: 0.6% vs. boys: 0.9%) |
|  | Al-Hussein et al. [17] | Elevated fasting blood sugar (≥6.1 mmol/l), 0.6% (girls: 0.9% vs. boys: 0.4%) |
| *Blood pressure* | Household Health Survey [10] | Hypertension, 0.7% |
|  | Moradi-Lakeh et al. [5] | Normal blood pressure (SBP ≤120 and DBP ≤80); 64.4%, pre-hypertension (120< SBP <140 or 80< DBP <90); 31.3%, Stage 1 hypertension (140≤ SBP <160 or 90≤ DBP <100), 3.9%; Stage 2 hypertension (SBP ≥160 or DBP ≥100), 0.4% |
| *Tobacco use* | Household Health Survey [10] | Current smoker, 2.1%, passive smokers (“second-hand” smokers), 25.8% |
|  | Moradi-Lakeh et al. [5] | Current smoker, 3.5%; former smoker, 2.1%; never smoked, 94.4%; sheesha (water pipe) daily, 1.5%, other, 98.5% |
|  | Al-Buhairan et al. [16] | Ever smoked cigarettes, 16.2% (girls: 9.6% vs. boys: 22.1%); sheesha (water pipe), 10.5% (girls: 7.1% vs. boys: 13.5%); solvent sniffing in the preceding month, 16% (girls: 21.4% vs. boys: 11.5%) |
| *PA and sedentary behavior* | Household Sports Practice Survey [18] | Practice sports activity (150 minutes and more per week), 23.8% (girls: 9.0% vs. boys: 38.0%) |
|  | Moradi-Lakeh et al. [5] | No PA, 25.0% (girls: 42.8% vs. boys: 19.7%); insufficient PA, 29% (girls: 32.8% vs. boys: 22.1%); moderate PA, 12.6% (girls: 9% vs. boys 16%); vigorous PA, 33.0% (girls: 15.4% vs. boys 42.1%), sedentary behavior, 4.5 (SE=0.1) hr (girls: 4.7 (0.1) vs. boys: 4.4 (0.1)) |
|  | Baqal et al. [6] | Engage in PA, 53.4% (girls: 40.7% vs. boys: 68.3%); engage in PA (sports) at school, 35.4%; no of d spent on >30 min exercise, 1.9 ± 2.4; television viewing (≥2 h/d), 42% (girls: 44.7% vs. boys: 40.4%) |
| *Dietary habits and nutrient deficiencies* | Household Health Survey [10] | Consumed the recommended intake of fruits and vegetables, 10% |
|  | Moradi-Lakeh et al. [5] | Fruit and vegetable intake: no servings/d, 37%, 1 to 4 servings/d, 57.2%, ≥5 servings/d, 5.3% |
|  | Al-Buhairan et al. [16] | Fruit intake (≥1 servings/d), 38% (girls: 31.8% vs. boys: 43.6%); vegetable intake (≥1 servings/d), 54.3% (girls: 52.8% vs. boys: 50.7%); carbonated beverage consumption (≥2 drinks/d), 37.5% (girls: 30.4% vs. boys: 43.9%); energy drinks consumption (≥1 drinks/d), 21.8% (girls: 17.7% vs. boys: 25.5%); vitamin D deficiency (<50 nmol/L), 95.6% |
|  | Al-Dakheel et al. [19] | Total goitre rate, 4.2% ( girls: 7.1%, boys: 3.1%); 3.6% for grade 1 and 0.6% for grade 2; prevalence was <5% in all parts of the country except the southern region, estimated at 12.7% |
| *Sleep habits* | Al-Hazzaa et al. [20] | Nocturnal sleep duration <9 h, 65.8% (girls: 64.4% vs. boys: 67.5%) |
|  | Nasim et al. [21] | Sleep deprivation (<7 h ) on weekdays, 45.7%; on weekends, 33.5% |
| *Road traffic safety* | Moradi-Lakeh et al. [5] | Always wore a seatbelt as a driver, 4.8%; never used a seatbelt as a driver, 60.6%; never used seatbelts as front passengers, 75.3%; always using a seatbelt, 2.8%; never used a seatbelt as back-seat passengers, 90.6%; always follow the speed limit 24.4%; never following the speed limit, 24.1%; using hands-free cellphones, 3.9% |
|  | Al-Buhairan et al. [16] | Sometimes/always wear seatbelt, 13.8%; drive a car without permission 17.9% |
|  | Household Health Survey [10] | Sustained road traffic injuries, 14,559 adolescents; injured in other accidents (not road traffic related), 21,679 adolescents |
| *Note*: Body mass index (BMI); Diastolic blood pressure (DBP); Physical activity (PA); Saudi Health Information Survey (SHIS); Systolic blood pressure (SBP); World Health Organization (WHO). 13 regions of Saudi Arabia (Al Riyadh, Makkah, Eastern Region, Northern Borders, Madinah, Jezan, Aseer, Najran, Qaseem, Tabuk, Hail, Al‐Jouf, Al‐Baha). 20 educational regions (Al Riyadh, Jeddah, Eastern Province, Makkah, Al-Madinah, Al-Qassim, Al-Taif, Al-Qurayyat, Al-Jouf, Tabouk, Najran, Jazan, Hail, Al-Bahaa, Aseer, Al-Ahsa, Al- Qunfudhah, Hafar Al-Batin, Bisha, and the Northern Borders)  a. Also known as the Periodic Examination Program for School Students (PEPSS). | | |

| **Table S3. Risk of bias assessment using the Newcastle-Ottawa scale for cross-sectional studies** | | | | | | | | | | |
| --- | --- | --- | --- | --- | --- | --- | --- | --- | --- | --- |
|  | **Selection (0-4)** | | | | **Comparability (0-2)** | **Outcome (0-3)** | |  |  |  |
| **Study** | **Representativeness of the sample** | **Sample size** | **Non-respondents** | **Ascertainment of the exposure** | **Comparability of subjects in different outcome groups on the basis of design or analysis** | **Assessment of outcome** | **Statistical test** | **Total score** | **Risk of bias** |  |
| AbouAbbas et al. [9] | 1 | 1 | 1 | — | 2 | — | 1 | 6 | Moderate |  |
| Al-Buhairan et al. [16] | 1 | 1 | 1 | — | 2 | 2 | 1 | 8 | Low |  |
| Al-Daajani et al. [1] | — | 1 | 1 | 2 | — | 2 | 1 | 7 | Low |  |
| Al-Dakheel et al. [19] | 1 | 1 | 1 | 2 | — | 2 | 1 | 8 | Low |  |
| Al-Ghamdi et al. [11] | — | — | — | 2 | 2 | 2 | 1 | 7 | Low |  |
| Al-Ghamdi et al. [12] | — | — | — | 2 | 2 | 2 | 1 | 7 | Low |  |
| Al-Hazzaa et al. [20] | — | 1 | — | — | 2 | — | 1 | 4 | High |  |
| Al-Hussein et al. [17] | — | 1 | — | 2 | — | 2 | 1 | 6 | Moderate |  |
| Al-Qahtani et al. [15] | — | 1 | — | 1 | 2 | — | 1 | 5 | Moderate |  |
| Baqal et al. [6] | 1 | 1 | 1 | — | — | — | 1 | 4 | High |  |
| Global Health Observatory data repository, WHO [3] | n.a. | n.a. | n.a. | n.a. | n.a. | n.a. | n.a. | n.a. | n.a. |  |
| Global Burden of Disease (GBD) data [7] | n.a. | n.a. | n.a. | n.a. | n.a. | n.a. | n.a. | n.a. | n.a. |  |
| Household Health Survey [10] | 1 | 1 | 1 | — | — | — | 1 | 4 | High |  |
| Household Sports Practice Survey [18] | 1 | 1 | 1 | — | — | — | 1 | 4 | High |  |
| Ibrahim et al. [4] | 1 | 1 | — | — | — | — | 1 | 3 | High |  |
| Moradi–Lakeh et al. [5] | 1 | 1 | 1 | — | — | — | 1 | 4 | High |  |
| Musharrafieh et al. [14] | 1 | 1 | 1 | — | 2 | — | 1 | 6 | Moderate |  |
| Nasim et al. [21] | 1 | 1 | 1 | — | 2 | — | 1 | 6 | Moderate |  |
| Shewear-AlAbdulrhman et al. [2] | — | 1 | 1 | 2 | — | 1 | 1 | 6 | Moderate |  |
| Saudi Health Interview Survey (SHIS) [13] | 1 | 1 | — | 1 | 2 | 2 | 1 | 8 | Low |  |
| Saudi Mental Health Survey (SNMHS) [8] | — | 1 | — | — | 2 | — | 1 | 4 | High |  |
| n.a. = not applicable. — = not available | | | | | | | | | |  |

| **Table S4. Egger’s regression tests for publication bias by indicator** | | | | | |
| --- | --- | --- | --- | --- | --- |
| **Indicator** | **Studies** | **Egger intercept** | **SE intercept** | **CI 95%** | **P value** |
| Overweight | 5 | -1.87 | 12.44 | -41.47, 37.74 | 0.89 |
| Obesity | 5 | 5.37 | 16.25 | -46.35, 57.09 | 0.76 |
| Mental health | 3 | 10.54 | 29.21 | -360.66, 381.73 | 0.78 |
| Dental health | 4 | 70.01 | 58.65 | -182.35, 322.37 | 0.35 |
| Asthma | 3 | 1.42 | 30.06 | -380.52, 383.36 | 0.97 |
| Elevated blood glucose | 4 | -7.37 | 3.42 | -22.09, 7.35 | 0.16 |
| High Blood pressure | 5 | 41.79 | 30.57 | -55.51, 139.08 | 0.27 |
| Tobacco use | 6 | -63.79 | 66.05 | -247.18, 119.60 | 0.39 |
| Physical activity and sedentary habits | 6 | -5.14 | 19.89 | -60.37, 50.10 | 0.81 |
| Dietary habits and nutrient deficiencies | 9 | 61.79 | 26.40 | -0.64, 124.22 | 0.05 |
| Sleep habits | 3 | 22.95 | 29.59 | -353.04, 398.94 | 0.58 |
| Road traffic safety | 5 | 53.72 | 22.49 | -17.85, 125.28 | 0.10 |

Abbreviations: CI, confidence interval, SE, standard error. Egger intercept, regression intercept from Egger’s test estimated on the logit scale of prevalence. p value, two sided. Results with p greater than 0.05 indicate no statistical evidence of small study effect.

**References**

1. Al Daajani, M.M., et al., *Prevalence of Health Problems Targeted by the National School-Based Screening Program among Primary School Students in Saudi Arabia, 2019.* Healthcare (Basel), 2021. **9**(10).

2. Shewear-Al Abdulrhman N, B.A.A., *Assessment of School Health in Saudi Arabia: The Path to Improved Future Students Health. (Implications of the Saudi School Health Program).* J Public Health Dis Prev, 2022. **5**: p. 102.

3. *WHO (World Health Organization). Global Health Observatory data repository. Prevalence of obesity among children and adolescents, by country. Geneva: WHO. Available from:* [*https://apps.who.int/gho/data/view.main.BMIPLUS2C05-09v?lang=en*](https://apps.who.int/gho/data/view.main.BMIPLUS2C05-09v?lang=en)*.*

4. Ibrahim, M., et al., *School-Based Body Mass Index Survey: A national study of Obesity Prevalence among School Students in Saudi Arabia, 2018: Obesity Prevalence among School Students in Saudi Arabia, 2018.* Journal of Health Informatics in Developing Countries, 2021. **15**(2).

5. Moradi-Lakeh, M., et al., *The health of Saudi youths: current challenges and future opportunities.* BMC Fam Pract, 2016. **17**: p. 26.

6. Baqal, O.J., H. Saleheen, and F.S. AlBuhairan, *Urgent Need for Adolescent Physical Activity Policies and Promotion: Lessons from "Jeeluna".* Int J Environ Res Public Health, 2020. **17**(12).

7. *IHME (Institute for Health Metrics and Evaluation). Global Burden of Disease Compare. Seattle, WA: IHME, University of Washington, 2015. Available from:* [*https://vizhub.healthdata.org/gbd-compare*](https://vizhub.healthdata.org/gbd-compare) *(Accessed [2 July 2022])*

8. *SNMHS (Saudi National Mental Health Survey). Saudi Arabia: King Salman Center for Disability Research.*

9. Abou Abbas, O. and F. AlBuhairan, *Predictors of adolescents' mental health problems in Saudi Arabia: findings from the Jeeluna(®) national study.* Child Adolesc Psychiatry Ment Health, 2017. **11**: p. 52.

10. *GASTAT (General Authority for Statistics). 2018. Household Health Survey. Saudi Arabia: GASTAT.* [*https://www.stats.gov.sa/en/965*](https://www.stats.gov.sa/en/965)*.*

11. AlGhamdi, A., et al., *Prevalence of periodontitis in high school children in Saudi Arabia: a national study.* Ann Saudi Med, 2020. **40**(1): p. 7-14.

12. AlGhamdi, A.S., et al., *Gingival health and oral hygiene practices among high school children in Saudi Arabia.* Ann Saudi Med, 2020. **40**(2): p. 126-135.

13. *IHME (Institute for Health Metrics and Evaluation), Ministry of Health (Saudi Arabia). Saudi Arabia Health Interview Survey 2013. Available from:* [*https://ghdx.healthdata.org/record/saudi-arabia-health-interview-survey-2013*](https://ghdx.healthdata.org/record/saudi-arabia-health-interview-survey-2013)*.*

14. Musharrafieh, U., et al., *A nationwide study of asthma correlates among adolescents in Saudi Arabia.* Asthma Res Pract, 2020. **6**: p. 3.

15. Alqahtani, J.M., *Asthma and other allergic diseases among Saudi schoolchildren in Najran: the need for a comprehensive intervention program.* Ann Saudi Med, 2016. **36**(6): p. 379-385.

16. AlBuhairan, F.S., et al., *Time for an Adolescent Health Surveillance System in Saudi Arabia: Findings From "Jeeluna".* J Adolesc Health, 2015. **57**(3): p. 263-9.

17. Al-Hussein, F.A., et al., *Cardiometabolic risk among Saudi children and adolescents: Saudi childrens overweight, obesity, and lifestyles (S.Ch.O.O.Ls) study.* Ann Saudi Med, 2014. **34**(1): p. 46-53.

18. *GASTAT (General Authority for Statistics). 2019. Household Sport Practice Survey Bulletin. Saudi Arabia: GASTAT.* [*https://www.stats.gov.sa/en/950-0*](https://www.stats.gov.sa/en/950-0)*.*

19. Al-Dakheel, M.H., et al., *Prevalence of iodine deficiency disorders among school children in Saudi Arabia: results of a national iodine nutrition study.* East Mediterr Health J, 2016. **22**(5): p. 301-8.

20. Al-Hazzaa, H.M., et al., *Anthropometric, Familial- and Lifestyle-Related Characteristics of School Children Skipping Breakfast in Jeddah, Saudi Arabia.* Nutrients, 2020. **12**(12).

21. Nasim, M., M. Saade, and F. AlBuhairan, *Sleep deprivation: prevalence and associated factors among adolescents in Saudi Arabia.* Sleep Med, 2019. **53**: p. 165-171.
